# Isolated Metalloid Tellurium Atomic Cluster on Nitrogen‐Doped Carbon Nanosheet for High‐Capacity Rechargeable Lithium‐CO_2_ Battery

**DOI:** 10.1002/advs.202205959

**Published:** 2023-01-16

**Authors:** Ke Wang, Dongyu Liu, Limin Liu, Xinyang Li, Hu Wu, Zongjie Sun, Mingtao Li, Andrey S. Vasenko, Shujiang Ding, Fengmei Wang, Chunhui Xiao

**Affiliations:** ^1^ Xi'an Key Laboratory of Sustainable Energy Materials Chemistry School of Chemistry Energy Storage Materials and Chemistry of Shaanxi University Engineering Research Center Xi'an Jiaotong University 28 Xianning West Road Xi'an Shaanxi 710049 China; ^2^ National Research University Higher School of Economics (HSE University) 20 Myasnitskaya Str. Moscow 101000 Russia; ^3^ International Research Center for Renewable Energy (IRCRE) State Key Laboratory of Multiphase Flow in Power Engineering (MFPE) Xi'an Jiaotong University 28 Xianning West Road Xi'an Shaanxi 710049 China; ^4^ State Key Laboratory of Chemical Resource Engineering Beijing Advanced Innovation Center for Soft Matter Science and Engineering Beijing University of Chemical Technology Beijing 100029 China

**Keywords:** amorphous discharge product, free‐standing electrode, metal‐free catalyst, metalloid catalyst, rechargeable Li‐CO_2_ battery

## Abstract

Rechargeable Li‐CO_2_ battery represents a sustainable technology by virtue of CO_2_ recyclability and energy storage capability. Unfortunately, the sluggish mass transport and electron transfer in bulky high‐crystalline discharge product of Li_2_CO_3_, severely hinder its practical capacity and rechargeability. Herein, a heterostructure of isolated metalloid Te atomic cluster anchored on N‐doped carbon nanosheets is designed (Te_AC_@NCNS) as a metal‐free cathode for Li‐CO_2_ battery. X‐ray absorption spectroscopy analysis demonstrates that the abundant and dispersed Te active centers can be stabilized by C atoms in form of the covalent bond. The fabricated battery shows an unprecedented full‐discharge capacity of 28.35 mAh cm^−2^ at 0.05 mA cm^−2^ and long‐term cycle life of up to 1000 h even at a high cut‐off capacity of 1 mAh cm^−2^. A series of ex situ characterizations combined with theoretical calculations demonstrate that the abundant Te atomic clusters acting as active centers can drive the electron redistribution of carbonate via forming Te—O bonds, giving rise to poor‐crystalline Li_2_CO_3_ film during the discharge process. Moreover, the efficient electron transfer between the Te centers and intermediate species is energetically beneficial for nucleation and accelerates the decomposition of Li_2_CO_3_ on the Te_AC_@NCNS during the discharge/charge process.

## Graphical abstract

A heterostructure of isolated metalloid Te atomic cluster anchored on N‐doped carbon nanosheet is designed as a metal‐free cathode for Li‐CO_2_ battery, which shows an unprecedented full‐discharge capacity of 28.35 mAh cm^−2^ at 0.05 mA cm^−2^ and long‐term cycle life of up to 1000 h even at a high cut‐off capacity of 1 mAh cm^−2^.

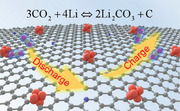

## 1 Introduction

Rechargeable aprotic lithium‐carbon dioxide (Li‐CO_2_) battery has triggered worldwide interest over the past decade owing to its environmentally friendly CO_2_ recyclability and sustainable energy storage system.^[^ ^1^ ^]^ A typical Li‐CO_2_ battery based on the reversible redox reaction of 4Li^+^+ 3CO_2_+ 4e^‐^↔2Li_2_CO_3_+ C (E^0^ = 2.80 V vs Li/Li^+^) provides an ultrahigh theoretical specific energy density (≈1876 Wh kg^−1^);^[^ ^2^ ^]^ this can make up for the low capacity of the current energy storage devices dominated by traditional lithium‐ion batteries (≈265 Wh kg^−1^) and promote the development of long‐distance electric vehicles and grid‐scale energy distributing systems.^[^ ^3^, ^4^ ^]^ Moreover, the Li‐CO_2_ battery is also expected to be extended to aerospace exploration, especially on Mars, where CO_2_ constitutes 96% of the atmospheric composition. Unfortunately, the intractable hurdles regarding large polarization and pitiful reversibility, originated from the electrochemically stable discharge product lithium carbonate (Li_2_CO_3_) in the cathode, overshadow the commercial viability of Li‐CO_2_ batteries.^[^ ^5^, ^6^, ^7^ ^]^ Although substantial strategies focused on constructing hierarchical porous architectures loaded with various electrocatalytic nanoparticles, such as carbon materials,^[^ ^8^, ^9^, ^10^ ^]^ metals and their compounds,^[^ ^11^, ^12^, ^13^ ^]^ metal‐organic framework compounds,^[^ ^14^, ^15^ ^]^ etc., have sprung up to accelerate the Li_2_CO_3_ decomposition during the charging process, the bulky insulting Li_2_CO_3_ usually accumulated on the cathode surface inhomogeneously and blocked the catalytic sites and gas diffusion channels, making the practical discharge capacities still far below the theoretical value and inferior cyclability.^[^ ^16^, ^17^ ^]^ Therefore, for high‐performance Li‐CO_2_ batteries, it is critically essential to design novel cathode catalysts for directing the uniform growth of amorphous discharge products instead of forming bulky high‐crystalline ones.

Tellurium (Te) as a typical metalloid element demonstrates exceptional superiorities embracing outstanding metallic conductivity and relatively strong CO_2_ adsorption,^[^ ^18^ ^]^ which is therefore expected to have potential catalytic activity for Li‐CO_2_ battery. In particular, isolated Te active centers can be easily stabilized by numerous C atoms in form of the covalent bond rather than a small quantity of N atoms in N‐doped carbons.^[^ ^19^, ^20^ ^]^ This would substantially increase the content of accessible active sites and ensure effective contact between the catalyst and Li_2_CO_3_/CO_2_. But a few pioneering works associated with dispersed metalloids only focus on aqueous electrocatalysis systems, such as hydrazine fuel cells^[^ ^19^ ^]^ and N_2_ electroreduction reactions.^[^ ^21^ ^]^ The straightforward modulation effect of metalloid active sites on CO_2_ reduction or CO_2_ evolution reaction of aprotic Li‐CO_2_ batteries is still in infancy.

In this work, we initially design a unique heterostructure with isolated metalloid Te atomic clusters anchored on N‐doped carbon nanosheets (Te_AC_@NCNS) via successive three‐step (i.e., gelatinization, freeze‐drying, and calcination) processes. When applying the Te_AC_@NCNS as the cathode catalysts, the Li‐CO_2_ battery shows an unprecedented full‐discharge capacity of 28.35 mAh cm^−2^ at 0.05 mA cm^−2^, remarkably decreased charge/discharge polarization (only 0.97 V at 0.025 mA cm^−2^) and comparable cyclability (e.g., 60 cycles at 0.05 mA cm^−2^, 120 cycles at 0.1 mA cm^−2^). More importantly, this battery can be reversibly discharged and charged for 25 cycles and work continuously for about 1000 h even at a high cut‐off capacity of 1 mAh cm^−2^, outperforming the mostly reported works. A series of structural characterizations combined with the theoretical calculations reveal that the Te atomic clusters functioning as catalytic centers could drive electron redistribution of discharge product Li_2_CO_3_ via forming Te—O bonds between Te and CO_3_^2−^, obstructing the Li_2_CO_3_ crystallization. The resulting large‐area and poorly crystalline discharge product could be reversibly decomposed by making full use of the intimately contacted catalytic centers, guaranteeing excellent recovery capability. And the efficient electron transfer between Te active sites and intermediate species could result in a low Li_2_CO_3_ nucleation/decomposition barrier on the Te_AC_@NCNS surface.

## 2 Results and Discussion

### 2.1 Synthesis and Characterizations of Te_AC_@NCNS

**Figure 1:**
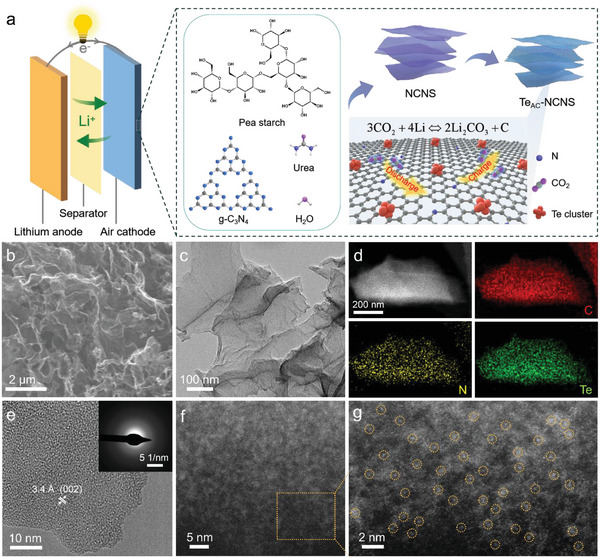
Synthesis and electron microscopy characterizations of Te_AC_@NCNS. a) The composition of the Li‐CO_2_ battery (left) and the scheme of the synthesis and the structure of the Te_AC_@NCNS (right). b) SEM image, c) TEM image, d) EDX elemental mapping image, and e) high‐resolution TEM image of the Te_AC_@NCNS. Inset: SAED pattern collected from e. f,g) HAADF‐STEM images of the Te_AC_@NCNS at different magnifications.

The Te_AC_@NCNS was prepared through the bottom‐up method (see Figure S1, Supporting Information and Experimental Section for details) with pea starch, g‐C_3_N_4_, and urea being used as the precursors (**Figure** **1**a). Typically, g‐C_3_N_4_ and urea were dispersed in the deionized water and heated to 100 °C under vigorous stirring. Then, the starch aqueous solution was dropped into the aforementioned mixture under continuous stirring to obtain the gel. The optical picture of the as‐synthesized hydrogel (Figure S2, Supporting Information) shows the large‐scale yield and uniformity. And the N‐doped carbon nanosheets (NCNS) were successfully synthesized through the freeze‐drying and pyrolysis processes. The carbonization yield of as‐prepared starch xerogel is determined to be ≈5.75 wt% according to the result of the thermo‐gravimetric analysis (TGA) (Figure S3, Supporting Information). Finally, the Te atomic clusters were anchored on the as‐synthesized NCNS via the chemical vapor deposition (see Figure S4 for details, Supporting Information). From scanning electron microscopy (SEM, Figure 1b) and transmission electron microscopy (TEM, Figure 1c; Figure S5, Supporting Information) images, both Te_AC_@NCNS and NCNS show a porous network‐like structure composed of ultrathin nanosheets with crumpled and wrinkled surfaces. The atomic force microscopy (AFM) image reveals the thickness of 6–10 nm for Te_AC_@NCNS (Figure S6, Supporting Information). In contrast with traditional carbon materials (e.g., carbon black), Te_AC_@NCNS would provide much more active areas for CO_2_ reduction and evolution reactions. The energy dispersive X‐ray (EDX) spectrum (Figure S7, Supporting Information) and corresponding element mapping images (Figure 1d) demonstrate the uniform dispersion of Te, N, and C in the Te_AC_@NCNS sample and the content of Te is 18.59 wt%. The high‐resolution TEM (HRTEM, Figure 1e) image collected from the edge of the nanosheets shows a lattice distance of 3.4 Å, corresponding to the (002) crystal plane of partially graphitized carbon. The selected area electron diffraction (SAED, inset of Figure 1e) confirms the poorly crystallized or amorphous nature of Te_AC_@NCNS. Furthermore, the high‐angle annular dark‐field scanning transmission electron microscopy (HAADF‐STEM, Figure 1f–g) images of the Te_AC_@NCNS nanosheet show the bright dots corresponding to Te atomic clusters across the entire NCNS backbone.

**Figure 2:**
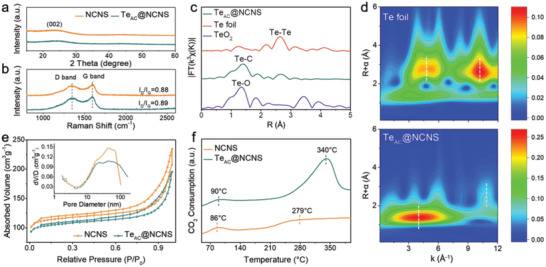
Structural Characterization of Te_AC_@NCNS and NCNS samples. a) XRD patterns, and b) Raman spectra of the Te_AC_@NCNS and NCNS nanosheets. c) Fourier‐transformed EXAFS spectra in *R* space for Te_AC_@NCNS, compared with those for Te foil and TeO_2_ references. d) WT‐EXAFS color map of Te_AC_@NCNS, compared with that of Te foil. e) N_2_ adsorption‐desorption isotherms and the pore size distribution (the inset) of Te_AC_@NCNS and NCNS samples. f) Normalized CO_2_‐TPD profiles of Te_AC_@NCNS and NCNS samples in the 50–400 °C.

Power X‐ray diffraction (XRD) patterns (**Figure** **2**a) collected from the NCNS and Te_AC_@NCNS samples show no obvious diffraction peaks except for a broad peak centered at 24.3°, which is attributed to the (002) reflections of graphitic carbon. Raman spectra (Figure 2b) of both samples reveal the D band located at 1350 cm^−1^ (sp^3^ hybridization and discorded defects) and the G band centered at 1596 cm^−1^ (sp^2^ hybridization and graphitic carbon). The intensity ratio between the D band and G band (I_D_/I_G_) for Te_AC_@NCNS (0.89) is almost equal to that of NCNS (0.88), reflecting a similar structural disorder. The X‐ray photoelectron spectroscopy (XPS) survey (Figure S8, Supporting Information) confirms the presence of C and N elements in both samples and the extra Te element in Te_AC_@NCNS. In particular, the high‐resolution C 1s spectrum (Figure S9, Supporting Information) shows a new peak ≈283.7 eV, which is assigned to the Te—C bond.^[^ ^22^ ^]^ Besides, both Te_AC_@NCNS and NCNS samples contain similar N content (4.9 at.%) with graphitic N (3.4 at.%, located at 400.9 eV) dominating over pyridinic N (1.5 at.%, located at 398.1 eV) (Figure S10, Supporting Information).^[^ ^23^ ^]^ To probe the coordination environment of Te atomic clusters in NCNS, X‐ray absorption spectroscopy (XAS) analysis was conducted. The Te *K*‐edge X‐ray absorption near‐edge structure (XANES) spectrum (Figure S11, Supporting Information) of Te_AC_@NCNS shows an absorption edge located higher than Te foil, indicating a more positively charged state of central Te atoms due to the lower electronegativity of Te (✗_Te_ = 2.1) than that of neighboring carbon atoms (✗_C_ = 2.5). The Fourier‐transformed extended X‐ray absorption fine structural (EXAFS) spectra (Figure 2c) of Te_AC_@NCNS show a clear Te—C bond (1.40 Å) and Te—Te bond (≈2.65 Å), suggesting the existence of carbon coordinated Te atomic clusters. In comparison, Te foil and TeO_2_ exhibit strong peaks at 2.64 Å and 1.34 Å at their EXAFS spectra, corresponding to Te—Te and Te—O bonds, respectively. Obviously, the Te—Te bond peak is much weak for Te_AC_@NCNS than that of Te foil owning to the small size of clusters and the inexistence of structure periodicity. The wavelet transforms (WT) of the Te *K*‐edge EXAFS spectrum for Te_AC_@NCNS (Figure 2d) also show two characteristic peaks of Te—C coordination at 4.16 Å^−1^ and Te—Te coordination at 10.84 Å^−1^. These characterizations provide solid proof for the existence of the Te atomic cluster anchored on the NCNS nanosheet.

Next, the N_2_ adsorption‐desorption isotherms (Figure 2e) of the NCNS and Te_AC_@NCNS samples show a typical hysteresis loop characteristic of a porous structure with a Brunaue‐Emmett‐Teller (BET) surface area of 455.35 and 416.46 m^2^g^−1^, respectively. The slightly decreased BET surface area of Te_AC_@NCNS could be attributed to the surface coverage and pore blocking of the NCNS caused by Te atomic clusters (see Table S1 for details, Supporting Information). The CO_2_ sorption isotherm measurement further reveals that the CO_2_ adsorption capacity of NCNS and Te_AC_@NCNS is 51.04 and 41.86 cm^3^ g^−1^, respectively (Figure S12, Supporting Information). The CO_2_ temperature‐programmed desorption (CO_2_‐TPD) was further carried out to estimate the binding strength between CO_2_ and catalysts (Figure 2f). Both Te_AC_@NCNS and NCNS samples exhibit two CO_2_ desorption peaks. The peaks at <100 °C are usually ascribed to the physisorbed CO_2_ molecules that are hardly activated during realistic reactions;^[^ ^24^ ^]^ while the desorption peaks above 100 °C are associated with the chemically adsorbed CO_2_ molecules. The enhanced peak of the Te_AC_@NCNS at higher temperatures (340 °C) compared with that of NCNS (279 °C), reflects that the introduction of Te atomic clusters can significantly improve the chemisorption ability_._ This is expected to promote the electron transfer between CO_2_ molecules and catalysts and boost the CO_2_ reduction reaction in the Li‐CO_2_ battery.^[^ ^25^ ^]^

### 2.2 Electrochemical Activity of the Li‐CO_2_ Battery with Te_AC_@NCNS Cathode

**Figure 3:**
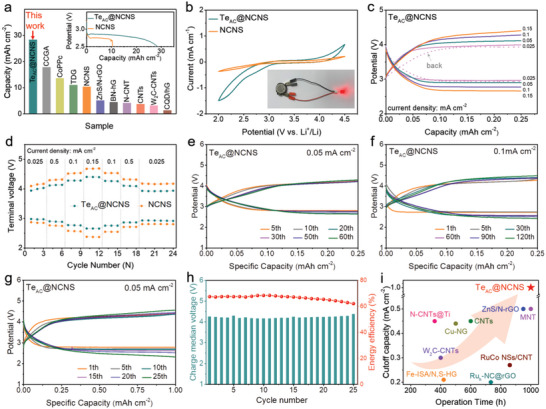
Electrochemical performance of Te_AC_@NCNS and NCNS‐based Li‐CO_2_ batteries. a) The comparison of the full‐discharge capacities with different cathodes. Inset: The first deep discharge curves at a current density of 0.05 mA cm^−2^. b) CV curves of the Te_AC_@NCNS and NCNS cathodes at 0.2 mV s^−1^ in CO_2_ atmosphere. Inset: The photograph of the assembled battery powering a light‐emitting diode. c) Discharge‐charge profiles of the Te_AC_@NCNS tested at various current densities. d) Comparison of the terminal voltage of discharge and charge for Te_AC_@NCNS and NCNS at different current densities. e) Discharge‐charge profiles of the Te_AC_@NCNS tested at 0.05 mA cm^−2^ with a cutoff capacity of 0.25 mAh cm^−2^. f) Discharge‐charge profiles of the Te_AC_@NCNS tested at 0.1 mA cm^−2^ with a cutoff capacity of 0.25 mAh cm^−2^. g,h) Cycling performance of Te_AC_@NCNS cathode at 0.05 mA cm^−2^ and a limited capacity of 1 mAh cm^−2^, and the corresponding median voltages of charge plateau and energy efficiency with the cycle number. i) Performance comparison diagram with different cathodes in terms of overpotential and operation time.

The elaborately designed two free‐standing samples were directly used as the cathodes of Li‐CO_2_ batteries to investigate their electrocatalytic activity. A homemade set‐up (Figure S13, Supporting Information) for providing a relatively stable CO_2_ atmosphere is used during all tests. The Te_AC_@NCNS‐based Li‐CO_2_ battery delivers a high full‐discharge capacity of 28.35 mAh cm^−2^ at a current density of 0.05 mA cm^−2^ (**Figure** **3**a), which outperforms the NCNS counterpart (10.379 mAh cm^−2^) and all reported cathode catalysts (1–17.9 mAh cm^−2^, Table S2, Supporting Information). We also tested the same battery in the Ar atmosphere and found that a negligible capacity is obtained, indicating that the discharge capacity does generate from CO_2_ reduction rather than other side reactions (Figure S14, Supporting Information). Notably, the Te_AC_@NCNS exhibits a higher discharge voltage than the pristine NCNS under the same conditions (inset of Figure 3a). These results imply that more CO_2_ catalytic sites and promoted reduction kinetics have been offered by the Te atomic clusters on the Te_AC_@NCNS cathode. Cyclic voltammetry measurements (Figure 3b) between 2.0 and 4.5 V (vs Li^+^/Li, the same hereafter) demonstrate that the Te_AC_@NCNS electrode possesses a strong capability of CO_2_ reduction/evolution with a more positive shift of onset potential for cathodic peak and more negative shift for the anodic peak compared with those of the NCNS, confirming the vital role of Te atomic clusters on enhancing CO_2_ reduction and evolution reaction kinetics. The inset of Figure 3b exhibits the photograph of the LED lighted by one specific Te_AC_@NCNS‐based Li‐CO_2_ battery, demonstrating its practical application potential.

The rate capabilities of the Li‐CO_2_ batteries were subsequently investigated by discharging and charging Te_AC_@NCNS and NCNS cathodes at different current densities, ranging from 0.025 to 0.15 mA cm^−2^. As can be seen in Figure 3c,d, the Te_AC_@NCNS exhibits steady discharge/charge plateaus and small discharge/charge voltage gaps at various current densities. Specifically, the Te_AC_@NCNS shows an extremely small voltage gap of 0.97 V at 0.025 mA cm^−2^, which is distinctly smaller than the value of 1.21 V for the NCNS (Figure 3d; Figure S15, Supporting Information). Even at an extremely high current density of 0.15 mA cm^−2^, the voltage gap of the Te_AC_@NCNS is only 1.74 V, whereas NCNS displays an overpotential of 2.31 V. This small voltage gap in rate performance is superior to those of previously reported Li‐CO_2_ batteries with the metal‐free and even some of the metal‐based catalysts (Table S2, Supporting Information). When the current density goes back to 0.025 mA cm^−2^, the discharge voltage of Te_AC_@NCNS shifts down a little and the charge voltage almost overlaps the original curve, demonstrating the outstanding rate capability.

Next, the long‐term cycling stability of the Li‐CO_2_ batteries was tested by continuously discharging and charging Te_AC_@NCNS and NCNS cathodes at a current density of 0.05 mA cm^−2^. As shown in Figure 3e, the Te_AC_@NCNS‐based battery can be reversibly discharged and charged at 60 cycles, and continuously work for at least 600 h. The median voltages of discharge/charge profiles have a little fluctuation at ≈2.79 V/≈4.02 V, corresponding to a low potential gap of 1.23 V. And the average energy efficiency is 76.0%, higher than the values collected from recently reported Li‐CO_2_ batteries.^[^ ^16^, ^26^ ^]^ For comparison, the NCNS cathode shows a much higher overpotential of 1.60 V (Figure S16, Supporting Information). When the current density is raised to 0.1 mA cm^−2^, the Te_AC_@NCNS‐based battery still possesses satisfactory durability of up to 600 h (120 cycles) and high energy efficiency of 68.73% (Figure 3f). Remarkably, the discharge voltage keeps at nearly 2.4 V at the end of 120 cycles, evidencing the fact that the activity of Te_AC_@NCNS could be well reserved after the cycling process; while the NCNS‐based battery only can deliver limited cycling stability of fewer than 11 cycles (Figure S17, Supporting Information). Even if the applied cutoff capacity is increased to 1 mAh cm^−2^, the Te_AC_@NCNS‐based battery can still be reversibly discharged and charged for 25 cycles and continuously work for about 1000 h (Figure 3h). Significantly, the corresponding charge media voltage keeps below 4.30 V and the energy efficiency keeps at an average of 66.0% during cycling (Figure 3i). The outstanding catalytic activity of Te_AC_@NCNS for Li‐CO_2_ batteries could be presented by comparison with some other proposed cathode materials, particularly according to the cutoff capacity and cycling time (Figure 3g; Table S2, Supporting Information).

### 2.3 Deeply Understanding the Discharge‐Charge Behavior of Te_AC_@NCNS Cathode

**Figure 4:**
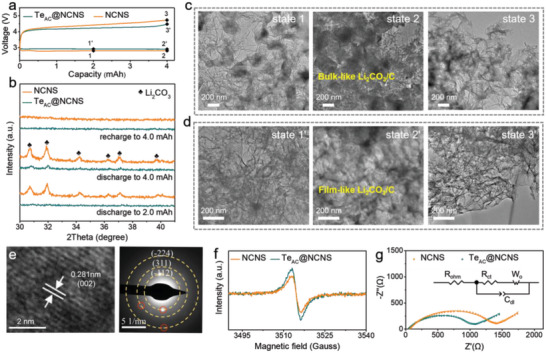
Ex situ characterizations of discharged and charged electrodes. a) Typical first discharge and charge curve of Li‐CO_2_ batteries with the Te_AC_@NCNS and NCNS as cathodes at the current density of 0.05 mA cm^−2^ with a terminal capacity of 4 mAh (≈5.10 mAh cm^−2^), and b) corresponding ex situ XRD patterns of Te_AC_@NCNS and NCNS cathodes at different discharge and charge states. TEM images of c) NCNS and d) Te_AC_@NCNS cathodes at different discharge and charge states. e) The periodic lattice spacing and selected area electron diffraction of discharged Te_AC_@NCNS cathode. f) ESR spectra, and g) electrochemical impedance spectra of discharged Te_AC_@NCNS and NCNS cathodes. The discharge states mentioned here are 4 mAh.

To deeply excavate the reason why the Te_AC_@NCNS cathode possesses such a satisfactory electrochemical performance, a series of ex situ techniques were performed to analyze the discharge products at discharge‐charge processes for as‐prepared electrodes. **Figure** **4**a,b presents the XRD profiles of Te_AC_@NCNS and NCNS cathodes at different stages during the discharge‐charge processes. As the discharge process proceeds, the peaks at 30.7°, 31.9°, 34.2°, 36.2°, 36.9°, and 39.6° belonging to Li_2_CO_3_ gradually emerge. Then these characteristic peaks of Li_2_CO_3_ disappear after the cell was fully recharged. Notably, the crystallinity of discharged Li_2_CO_3_ product considerably deferens for the as‐prepared two cathodes. Distinct diffraction peaks corresponding to Li_2_CO_3_ species are detected on the NCNS cathode discharged to 2.0 mAh. Similar to previously reported cases of Li_2_O_2_ in Li‐O_2_ batteries, sharp diffraction peaks usually indicate the formation of large crystal domains and the poor conductivity of the discharge products.^[^ ^27^, ^28^, ^29^ ^]^ On the contrary, for the Te_AC_@NCNS cathode, no peaks associated with Li_2_CO_3_ could be observed after discharge to the same capacity of 2.0 mAh, suggesting the amorphous nature of the discharge product. Even it was further discharged to 4.0 mAh, the diffraction peaks of Li_2_CO_3_ are still less intense than those of the NCNS cathode. All those results demonstrate that the usage of metalloid Te_AC_@NCNS could induce the formation of amorphous discharge products, especially at a relatively low depth of discharge. Similar findings have previously been reported on the noble metals of Ru‐based mobile catalysts.^[^ ^30^ ^]^

Moreover, the morphology of the discharge product can also be manipulated by the highly dispersed Te atomic clusters. Figure 4c,d show the TEM images of NCNS and Te_AC_@NCNS catalyst at different discharge and charge states, respectively, and the morphology of the discharge products are dramatically different even with the same current density of 0.05 mA cm^−2^. On the NCNS cathode, some bulk‐like product is discretely scattered over the electrode surface after being discharged to 2 mAh (state 1), where the NCNS nanosheet structure can still be observed. On subsequent discharge to 4 mAh (state 2), both the density and thickness of the discharge products increase, and the catalyst surface is almost covered by the densely stacked discharge product. This densely crystalline layer would separate reaction intermediates from contacting electrons/ions and then cause sluggish redox reaction kinetics.^[^ ^31^ ^]^ As expected, a small amount of discharge product remains on the nanosheet surface after recharging (state 3). Differently, when the Te_AC_@NCNS was used as the cathode, discharge products with a film‐like structure uniformly grow on the surface of Te_AC_@NCNS (state 1’). Further discharging the batteries to 4 mAh (state 2’), larger‐area film along the nanosheet is still observed. The sufficient contact interface between discharge products and the catalysts could take full advantage of the active centers of Te_AC_@NCNS, guaranteeing that the charging process is more energetically feasible. The corresponding periodic lattice spacing and selected area electron diffraction also show that the main discharge product is Li_2_CO_3_, consistent with the aforementioned XRD analysis (Figure 4e). On subsequent charge to 4 mAh (state 3’), the Li_2_CO_3_ is mostly decomposed to produce a clean surface for Te_AC_@NCNS. The electron spin resonance (ESR, Figure 4f) spectrum of the discharged Te_AC_@NCNS cathodes at 4 mAh exhibit a strong signal peak with *g* factor of 2.003, suggesting the existence of abundant unpaired electrons and defects in it.^[^ ^32^ ^]^ Compared with its crystalline siblings, the amorphous discharge product always demonstrates enhanced charge‐transport properties and increased electrooxidation kinetics.^[^ ^29^ ^]^ Meanwhile, the Nyquist plot (Figure 4g) of this discharged Te_AC_@NCNS exhibits smaller cathode/electrolyte interfacial resistance (40.5 Ω) and charge transport resistance (995.4 Ω) than those (54.1 and 1327.0 Ω, respectively) of discharged NCNS.

**Figure 5:**
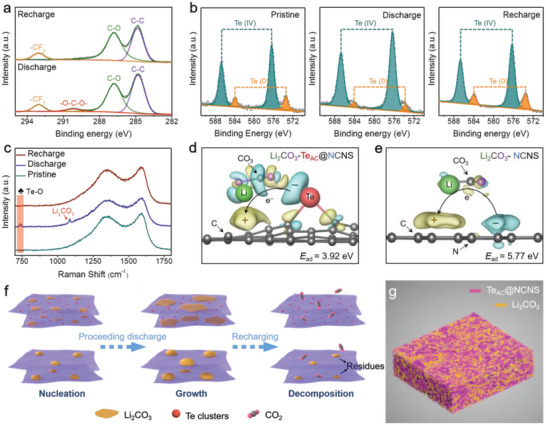
The reaction mechanism of Te_AC_@NCNS during the discharge‐charge cycling. a) The high‐resolution C 1s XPS spectra of Te_AC_@NCNS at discharge and recharge states. b) The high‐resolution Te 3*d* XPS spectra, and c) Raman spectra of Te_AC_@NCNS cathode at pristine, discharge, and recharge states. The discharge and charge states mentioned here are 4 mAh. d,e) Calculated charge density differences of Li_2_CO_3_ adsorption on Te_AC_@NCNS (isovalue = 0.002) and NCNS (isovalue = 0.001) with the adsorption energies. The blue and yellow regions indicate electron loss and gain, respectively. f) Schematic illustrations and comparison of the working mechanism of the Te_AC_@NCNS and NCNS cathodes. g) The 3D X‐ray tomography plot conducted on the Te_AC_@NCNS cathode after discharging to 10.0 mAh cm^−2^.

Then, ex situ XPS and Raman spectroscopy were performed to explore the surface state change of Te_AC_@NCNS during cycling. As shown in the high‐resolution C 1s XPS spectra (**Figure** **5**a), a new peak at ≈290.3 eV attributable to the O—C—O bond of Li_2_CO_3_ is only observed after discharge. Another augmented peaks of C—O at 286.8 eV and ‐CF_3_ at 293.1 eV originate from the electrolyte.^[^ ^16^, ^31^ ^]^ The fine Te 3D spectra of Te_AC_@NCNS show the typical Te 3*d*_5/2_ and Te 3*d*_3/2_ peaks, where the binding energies of 583.97 eV and 573.59 eV are assigned to the nonmetallic Te^0^, and the binding energies of 586.69 and 576.29 eV are attributed to the inevitable oxidized Te^IV^ (Figure 5b).^[^ ^33^, ^34^ ^]^ Note that the proportion of Te^0^ (low valence states) decreases rapidly while the Te^IV^ (high valence states) increases due to the continuous oxidation of Te^0^ to Te^IV^, suggesting that the Te active center could act as an electron donor during the discharge process. In addition, the O—C—O bond of Li_2_CO_3_ for discharged Te_AC_@NCNS has a slight shift to high binding energy compared to that of discharged NCNS (Figures S18 and S19, Supporting Information), implying again the strong electron transfer between Te atomic clusters and discharge product Li_2_CO_3_. After the recharge process, the discharge product Li_2_CO_3_ is fully decomposed, and the relative ratio of Te^4+^ and Te^0^ is returned to the original state. Similar results could be obtained from the Raman spectroscopy of the Te_AC_@NCNS at pristine, discharged, and recharged states. As shown in Figure 5c, except for the vibration peak of Li_2_CO_3_ (1087 cm^−1^), a new peak at 742 cm^−1^ appears after discharging, which is attributed to the Te—O vibrations^[^ ^35^, ^36^ ^]^ and vanishes after subsequently recharging. This phenomenon was not observed in the Raman spectrum of the discharged NCNS (Figure S20, Supporting Information), which only exhibits an extra peak related to the Li_2_CO_3_. Density functional theory (DFT) calculations were further performed to reveal the adsorption configurations of Li_2_CO_3_ on Te_AC_@NCNS and NCNS surface models and the corresponding charge density differences (Figures S21 and S22, Supporting Information). As shown in Figure 5d, significant charge transfer from Te to Li_2_CO_3_ through Te—O interaction and electron redistribution within CO_3_^2−^ are observed. In the contrast, NCNS only leads to slight charge transfer near the surface without interfering the Li_2_CO_3_ coordination (Figure 5e). Considering the CO_3_^2−^ species mainly bond with Li^+^ ion through the electrostatic force in Li_2_CO_3_, such Te—O interaction is expected to obstruct the lithium carbonate crystallization by disrupting its internal electron distribution. The much smaller adsorption energy (3.92 eV) indicates an enhanced interaction between Te atomic clusters and Li_2_CO_3_ compared with on NCNS cathode (5.77 eV).

Based on experimental results and computational analysis, a plausible route of discharge product growth and decomposition process for the NCNS and Te_AC_@NCNS catalysts is hypothesized (Figure 5f). Typically, during the CO_2_ reduction process, dissolved CO_2_ molecules undergo a four‐electron transfer reduction forming discharge products of Li_2_CO_3_ and amorphous C (4Li^+^+ 3CO_2_+ 4e^‐^ → 2Li_2_CO_3_+ C). Due to the relatively strong constraint effect from the higher affinity between Li_2_CO_3_ and Te centers, immobilized Li_2_CO_3_ is more inclined to anchor on the Te_AC_@NCNS surface without incorporation. The rich Te active sites can drastically disrupt the electron distribution of Li_2_CO_3_ and thus hinder its crystallization. With the increase of discharge depth, large‐area, and low‐crystalline discharge product films can be homogeneously and intimately deposited around Te active sites, establishing a low‐impedance Li_2_CO_3_/catalyst contact interface. During the recharge process, the unique discharge product structure taking advantage in ease of decomposition could make full use of the intimately contacted catalytic centers of Te atomic clusters, accelerating the CO_2_ evolution reaction kinetics and guaranteeing excellent reversibility. In sharp contrast, for the NCNS cathodes, owing to the relatively weaker binding interactions with Li_2_CO_3_ and limited accessible active sites, the discharge products tend to accumulate on the cathode surface and form large‐size aggregates, hindering the reaction intermediates from contacting with ions/electrons. In the end, the bulky crystalline Li_2_CO_3_ cannot be effectively oxidized during recharge, resulting in discharge product residues and inferior electrochemical performance. The detailed reconstruction of the whole discharged Te_AC_@NCNS cathode at the nanoscale was further observed through X‐ray tomography (Figure 5g; Figure S23, Supporting Information). The active material (pink) is partially covered by the discharge product (yellow) in a widely dispersed fashion, showing the large active area associated with the Te_AC_@NCNS architecture for the redox reactions during the discharge and charge process.

**Figure 6:**
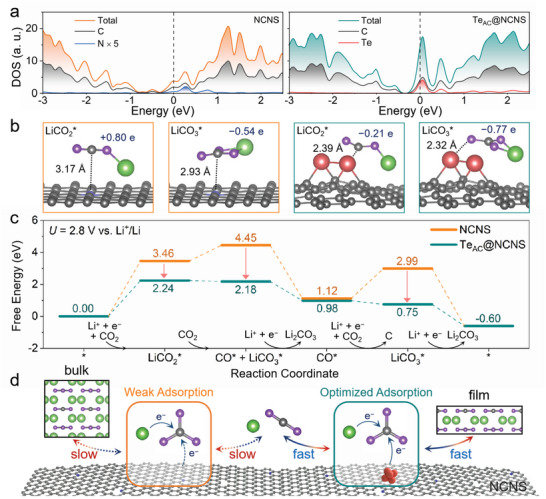
DFT calculations and proposed mechanisms. a) Total and element‐projected density of states (DOS) of NCNS and Te_AC_@NCNS. The highest occupied energy level is set to zero. b) Adsorption configurations of LiCO_2_* and LiCO_3_* on different surfaces. The insert numbers indicate the amount of charge transferred from surface to the adsorbate and their distance along the dash line in Å. Color code: grey for C, purple for O, green for Li, blue for N, and red for Te. c) Reaction free energy diagrams of the discharge process on the NCNS and Te_AC_@NCNS at 2.8 V versus Li^+^/Li. d) Illustration of the proposed reaction mechanism on different catalysts.

The DFT calculations were carried out to further explore the mechanism of the catalytic activity improvement contributed by Te atomic clusters. The density of states (DOS) of NCNS and Te_AC_@NCNS were calculated and compared in **Figure** **6**a. The Te_AC_@NCNS system displays a significant peak near the Fermi level in the DOS plot, suggesting a much more favorable capability of charge transfer. Figure 6b shows the adsorption configurations of crucial LiCO_2_* and LiCO_3_* intermediates (* represents the adsorbed state or adsorption site) on the NCNS and Te_AC_@NCNS surfaces. Based on the coordination distances between the catalyst surface and the adsorbate, we notice the introduction of Te atomic clusters efficiently enhances the adsorption of LiCO_2_* and LiCO_3_* (3.17 Å for LiCO_2_* on NCNS to 2.39 Å on Te_AC_@NCNS, and 2.93 Å for LiCO_3_* on NCNS to 2.32 Å on Te_AC_@NCNS). Meanwhile, the charge transferred, that is, 0.21 and 0.77 e, from the Te_AC_@NCNS surface to LiCO_2_* and LiCO_3_* respectively is increased as well. This indicates that the Te atomic clusters on NCNS can provide more charge than pure NCNS to stabilize LiCO_2_* and LiCO_3_* on the Te_AC_@NCNS surface. Figure 6c presents the reaction free energy diagram of the discharge process on Te_AC_@NCNS and NCNS surfaces at the equilibrium potential. For the NCNS surface model, the reaction is mainly limited by the formation of LiCO_2_* and LiCO_3_* with large free energy changes. The Te atomic clusters can enhance the interactions with these two intermediates as evidenced in Figure 6b. This effect dramatically reduces the free energy changes (see the red arrows in Figure 6c), and thus promotes the discharge reaction. Meanwhile, since the charging process shares some intermediates with the discharge process, the Te atomic clusters are also anticipated to facilitate the CO_2_ evolution reaction. Our calculation results demonstrate that the Te atomic clusters can reduce the barrier of the discharge and charge processes by adjusting the intermediate adsorption strength, and the corresponding mechanism is illustrated in Figure 6d. The accelerated discharge and charge processes would enable the Li‐CO_2_ battery cycle with fewer polarization energy losses.

## 3 Conclusion

In summary, we constructed isolated metalloid Te atomic cluster anchored on N‐doped carbon nanosheets (Te_AC_@NCNS) as cathode catalyst for Li‐CO_2_ battery. Structural characterizations combined with the theoretical calculations show that rich Te atomic clusters functioning as catalytic centers can drive the electron redistribution of discharge product Li_2_CO_3_ via forming Te—O bonds, hindering the crystallization of Li_2_CO_3_. Different from NCNS cathode, large‐area, and poorly crystalline discharge products are generated on the Te_AC_@NCNS cathode during the discharge process. Such uniformly distributed film‐like Li_2_CO_3_ can be easily decomposed by making full use of the abundant Te atomic clusters during charge process, substantially accelerating Li_2_CO_3_ decomposition kinetics. As a result, the Li‐CO_2_ battery based on the Te_AC_@NCNS cathode affords an unprecedented full‐discharge capacity of over 28.35 mAh cm^−2^ at the current density of 0.05 mA cm^−2^, and long‐term stable cycle life up to 1000 h even at a high cut‐off capacity of 1 mAh cm^−2^. This work would open an avenue for the application of atomically dispersed metalloid catalysts in the field of metal‐air batteries.

## 4 Experimental Section

### Material Characterization

The morphologies and microstructure of all samples were observed by FESEM (Gemini SEM 500) and TEM (Thermo Fisher Talos F200X). The detailed atom structures were obtained by HAADF‐STEM (JEOL JEM‐ARM200F, 200 kV) and XAS (Beijing Synchrotron Radiation Facility). The crystal structure was characterized by XRD (Bruker D8 Advance diffractometer). A Raman spectrometer (Renishaw inVia Qontor) was used to obtain the Raman spectra with a wavelength of 532 nm as the excitation source. XPS (Thermo Fisher ESCALAB Xi+) was performed to study the chemical properties of the elements. The specific surface area and the pore size distribution was obtained through the nitrogen adsorption/desorption method (ASAP 2020 Plus HD88 physisorption analyzer). TGA was implemented using a METTLER TOLEDO TGA/DSC3+ from room temperature up to 800 °C with a heating rate of 5 °C min^−1^ in an argon atmosphere. The CO_2_‐TPD was conducted on a Micromeritics AutoChem II 2920 instrument.

### Synthetic Procedures for g‐C_3_N_4_

The g‐C_3_N_4_ was synthesized according to the previous literature.^[^ ^37^ ^]^ Specifically, 20 g of urea was added into a quartz container with a cover, and then calcined in air at 550 °C for 2 h with a ramping rate of 5 °C min^−1^.

### Synthetic Procedures of Free‐Standing NCNS Catalysts

The synthesis procedure of NCNS catalysts was modified based on the previous literature.^[^ ^38^ ^]^ Specifically, 0.5 g of urea and 0.3 g of g‐C_3_N_4_ were dispersed in 40 mL of deionized water and heated to 100 °C. Then, 2 g starch dispersed in 10 mL deionized water was dropped into the former solution under vigorous stirring. We can observe that the mixture solution gradually thickened after 5 min. Then the viscous solution was poured into a Petri (90 mm in diameter) and cooled to room temperature. After freeze‐drying, the xerogel was obtained. The prepared xerogel was divided into the desired size (19 mm in diameter) and heated in the Ar atmosphere at 800 °C for 2 h with a ramping rate of 5 °C min^−1^ to obtain NCNS. This production could be easily scaled up to 5–10 folds.

### Synthetic Procedures of Free‐Standing Te_AC_@NCNS Catalysts

The Te_AC_@NCNS was synthesized through a high‐temperature process in tellurium vapor. Briefly, NCNS and excess Te powder were separately put into both ends of a quartz boat, and heated to 600 and 800 °C for 2 h under a mixed H_2_/Ar flow with 5 vol% H_2_, respectively (see Figure S4 for details, Supporting Information). Both ends were set to take the same minutes to reach the maximum temperature.

### Electrochemical Measurements

The electrochemical performances of all Li‐CO_2_ batteries were tested in a coin‐type cell with holes in a positive shell. Freestanding Te_AC_@NCNS and NCNS (10 mm in diameter) were directly used as cathodes. The lithium metal (diameter: 16 mm, thickness: 0.6 mm), glass fiber separator (GF/D Whatman), and 1 m LiTFSI in TEGDME were used as the anode, separator, and electrolyte, respectively. All batteries were assembled in an argon‐filled glove box (oxygen and water contents were <0.1 ppm). Before testing, the cells were placed in the CO_2_‐filled homemade chamber (Figure S12, Supporting Information) to allow stabilization for 12 h.

### DFT Calculations

Periodic DFT calculations were performed with Vienna Ab initio Simulation Package (VASP)^[^ ^39^, ^40^, ^41^, ^42^ ^]^ using the projected augmented‐wave (PAW) method.^[^ ^43^, ^44^ ^]^ The Perdew‐Burke‐Ernzerhof (PBE) exchange‐correlation functional^[^ ^45^ ^]^ and the plane wave basis set with the cutoff energy of 450 eV were employed to solve the Kohn‐Sham equation. The convergence criteria of electron and ion optimizations were set to 10^−5^ and 0.02 eV Å^−1^, respectively. A Gaussian smearing with *σ* of 0.05 eV was used. London dispersion forces were included using the D3 approach with Becke‐Jonson damping.^[^ ^46^, ^47^ ^]^ The carbon nanosheet substrate was represented by a 6 × 3$\sqrt{3}$ graphene supercell with 72 C atoms separated by 20 Å vacuum region. The NCNS and Te_AC_@NCNS models were constructed by replacing one C with one N and two C with two Te, respectively. The energy of C and Li_2_CO_3_ species were derived from bulk graphite and lithium carbonate models. The Brillouin zone was sampled using a 2 × 2 × 1 Monkhorst‐Pack type *k*‐point grid. The atomic charge population was calculated with the Bader analysis method.^[^ ^48^ ^]^ The thermodynamic corrections were performed with VASPKIT code^[^ ^49^ ^]^ and the structures were visualized with VESTA software package.^[^ ^50^ ^]^

## Conflict of Interest

The authors declare no conflict of interest.

## Supporting information

Supporting Information

## Data Availability

The data that support the findings of this study are available in the supplementary material of this article.
