# Supplementary material for: Isolated Metalloid Tellurium Atomic Cluster on Nitrogen‐Doped Carbon Nanosheet for High‐Capacity Rechargeable Lithium‐CO2 Battery
Source: Adv Sci (Weinh). 2023 Jan 16;10(7):2205959. doi: 10.1002/advs.202205959 (PMC9982571; doi:10.1002/advs.202205959)
Supplement: Supplementary file 1 — Supporting Information [file ADVS-10-2205959-s001.pdf]

ADVANCED SCIENCE
Open Access

Supporting Information

for *Adv. Sci.*, DOI 10.1002/advs.202205959

Isolated Metalloid Tellurium Atomic Cluster on Nitrogen-Doped Carbon Nanosheet for High-Capacity Rechargeable Lithium-CO_2 Battery

Ke Wang, Dongyu Liu, Limin Liu, Xinyang Li, Hu Wu, Zongjie Sun, Mingtao Li, Andrey S. Vasenko, Shujiang Ding, Fengmei Wang and Chunhui Xiao**

Isolated Metalloid Tellurium Atomic Cluster on Nitrogen-Doped Carbon Nanosheet for High-Capacity Rechargeable Lithium-CO_2 Battery

Ke Wang[1]‡, Dongyu Liu[2] ‡, Limin Liu[1]‡, Xinyang Li[1], Hu Wu[1], Zongjie Sun[1], Mingtao Li[3], Andrey S. Vasenko[2], Shujiang Ding[1], Fengmei Wang[4,] *, Chunhui Xiao[1,] *

[1] Xi'an Key Laboratory of Sustainable Energy Materials Chemistry, School of Chemistry, Xi'an Jiaotong University, 28 Xianning West Road, Xi'an, Shaanxi 710049, China

[2] National Research University Higher School of Economics (HSE University), 20 Myasnitskaya Str., Moscow, 101000, Russia

[3] International Research Center for Renewable Energy (IRCRE), State Key Laboratory of Multiphase Flow in Power Engineering (MFPE), Xi'an Jiaotong University, 28 Xianning West Road, Xi'an, Shaanxi 710049, China

[4] State Key Laboratory of Chemical Resource Engineering, Beijing Advanced Innovation Center for Soft Matter Science and Engineering, Beijing University of Chemical Technology, Beijing, 100029 China

‡ These authors contributed equally to this work.

*Corresponding E-mail: chunhuixiao@xjtu.edu.cn, wangfm@buct.edu.cn

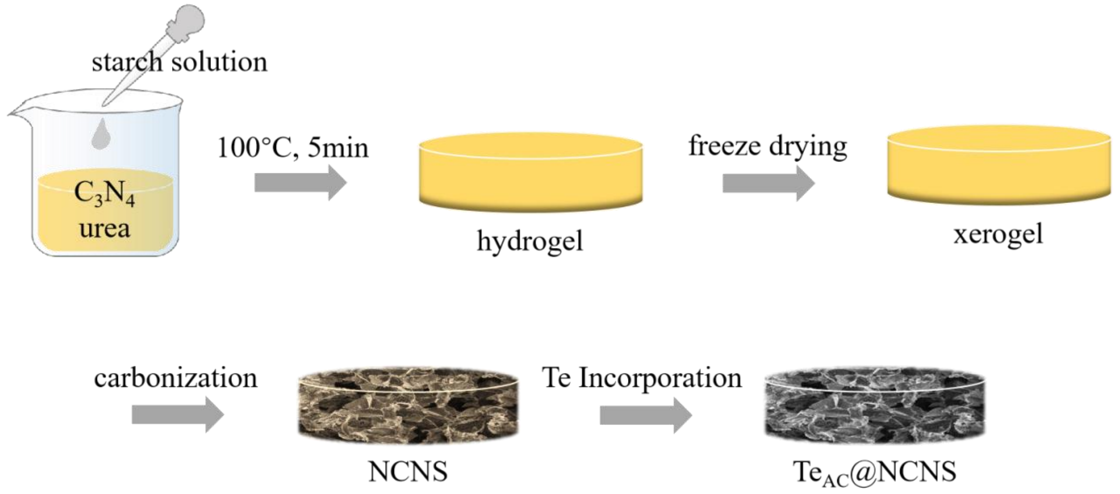

Figure S1. Schematics illustrating the detailed preparation procedure of the Te_{AC}@NCNS and NCNS.

It is well known that the starch is polymerized by glucose molecules with abundant hydroxyl groups, which can promote the supramolecular associations through inter and intramolecular hydrogen bonding. Upon heating to 100°C, starch granules absorb a large amount of water and expand rapidly, and the starch solution would become a viscous gel. In this process, layer-structured g-C_3N_4 functioned as a 2D template confines starch-derived carbon into their interlayers to form nanosheets, while urea served as a common foaming reagent releases numerous N-containing gases during the subsequent carbonization process to form N atoms in the carbon lattices and simultaneously guarantees the formation of the porous structure.

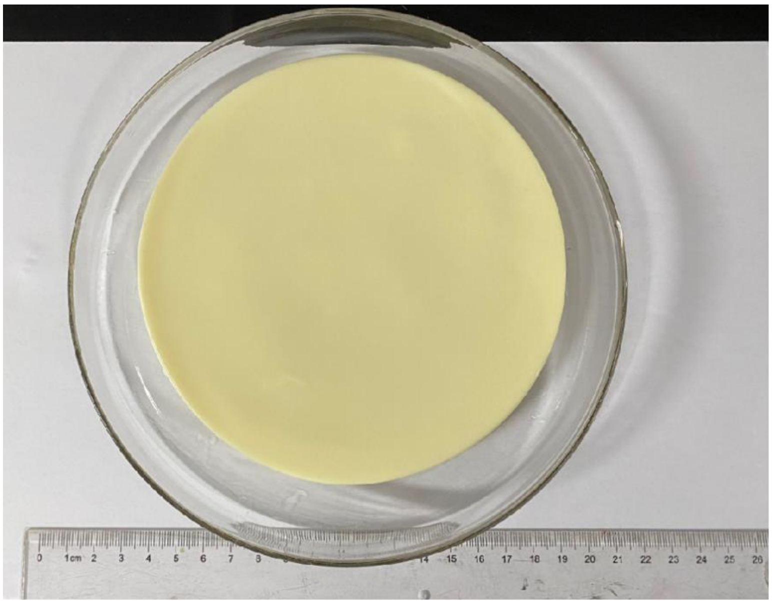

Figure S2. Optical photograph of the prepared hydrogel.

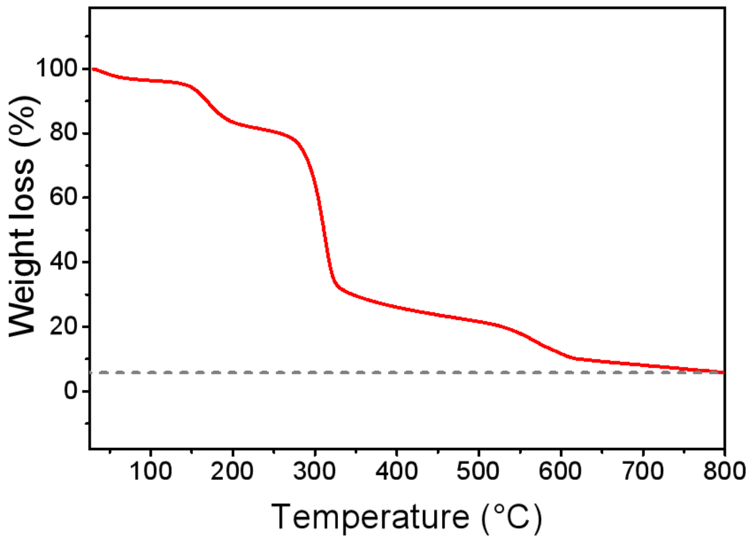

Figure S3. TGA curves of as-prepared xerogel.

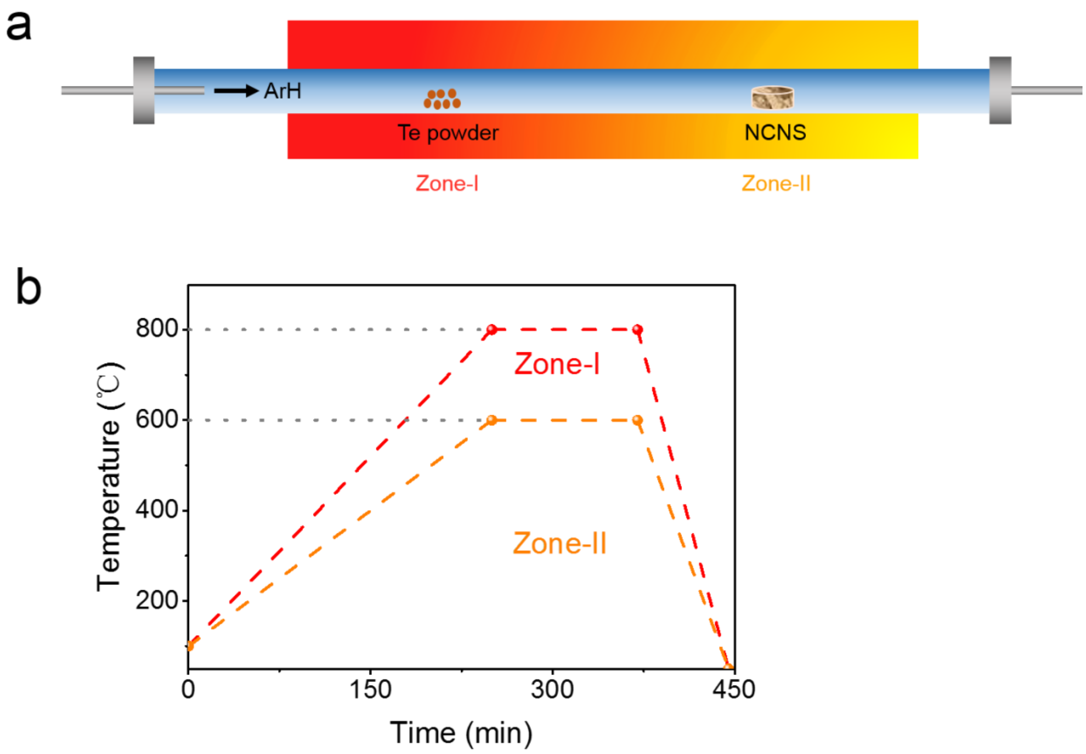

Figure S4. (a) Schematic diagram of set-up for Te incorporation. (b) The temperature profile for obtaining the Te_{AC}@NCNS.

In principle, with the processing temperature increasing, more graphitic N could incorporated into the carbon networks but the content of total N in as-obtained samples would decrease[1]. In order to avoid the large discrepancies for the content and types of N dopants in the Te_{AC}@NCNS and NCNS, as well as ensure sufficient Te vapor to participating in reaction, the processing temperature is divided into two regions.

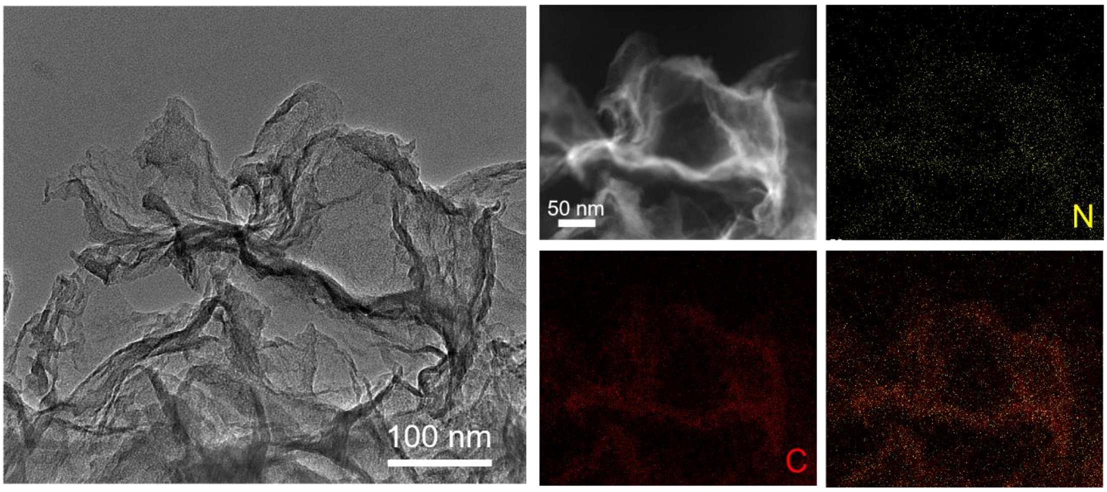

Figure S5. TEM image and corresponding elemental mappings of the NCNS.

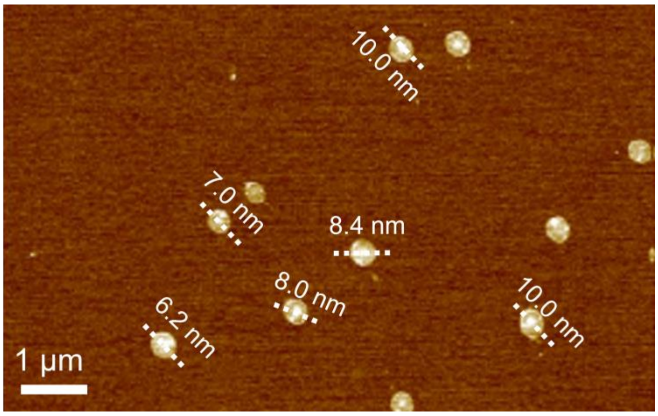

Figure S6. AFM height micrograph of the Te_{AC}@NCNS.

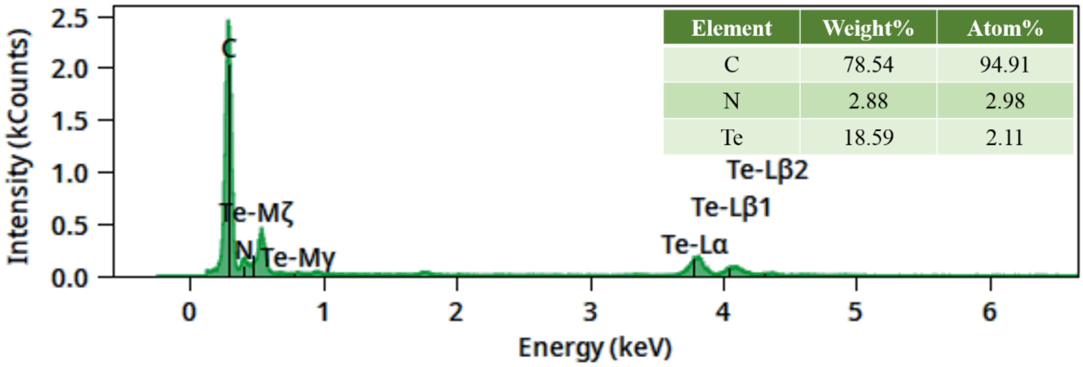

Element	Weight%	Atom%
C	78.54	94.91
N	2.88	2.98
Te	18.59	2.11

Figure S7. EDX spectrum of Te_{AC}@NCNS.

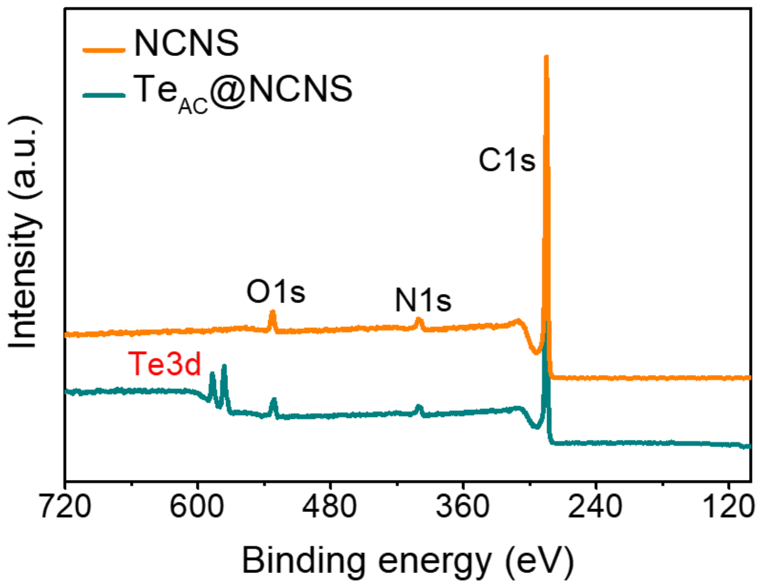

Figure S8. XPS surveys of the Te$_{AC}$@NCNS and NCNS.

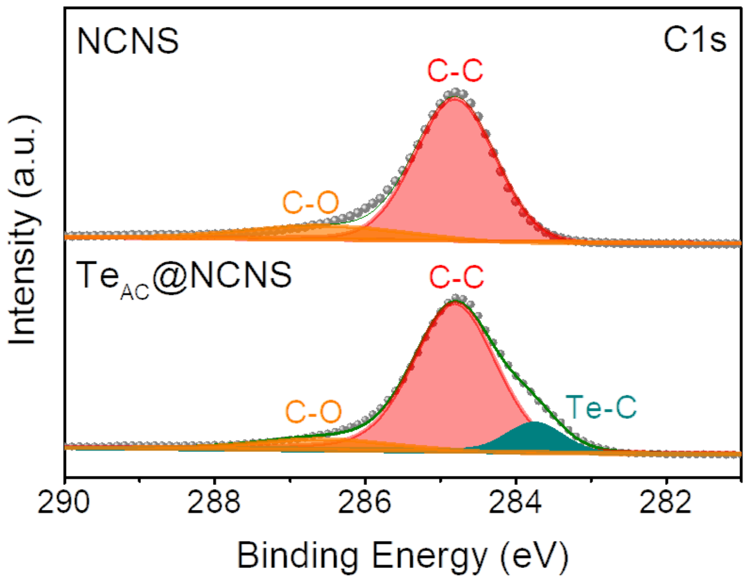

Figure S9. The high-resolution C 1*s* of the Te$_{AC}$@NCNS and NCNS.

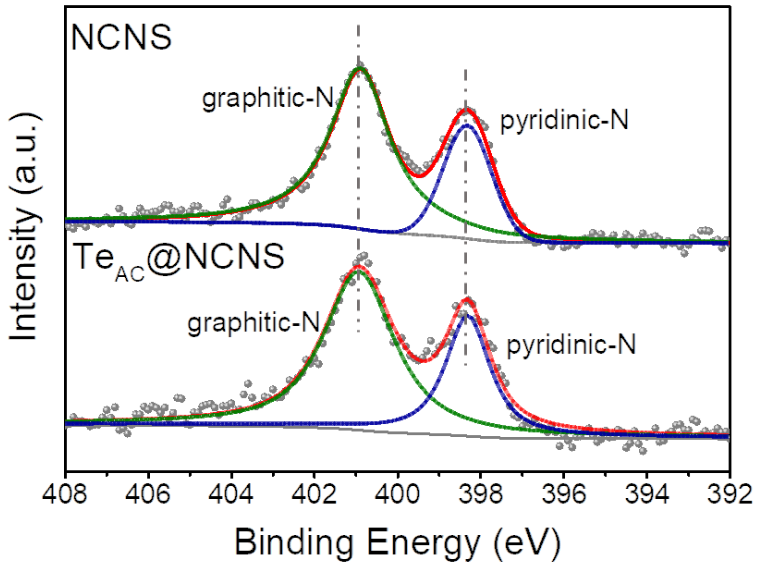

Figure S10. The high-resolution N 1*s* of the Te$_{AC}$@NCNS and NCNS.

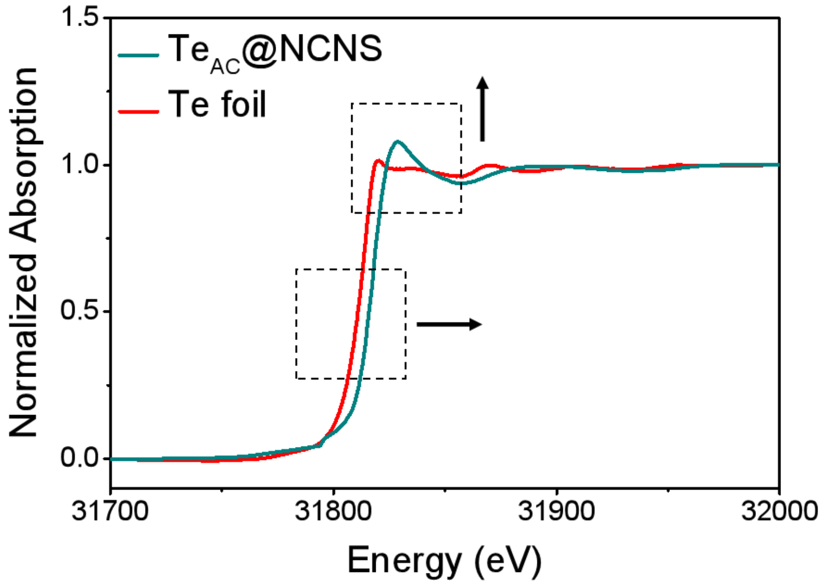

Figure S11. The Te *K*-edge X-ray absorption near-edge structure (XANES) spectrum of Te$_{AC}$@NCNS and Te foil.

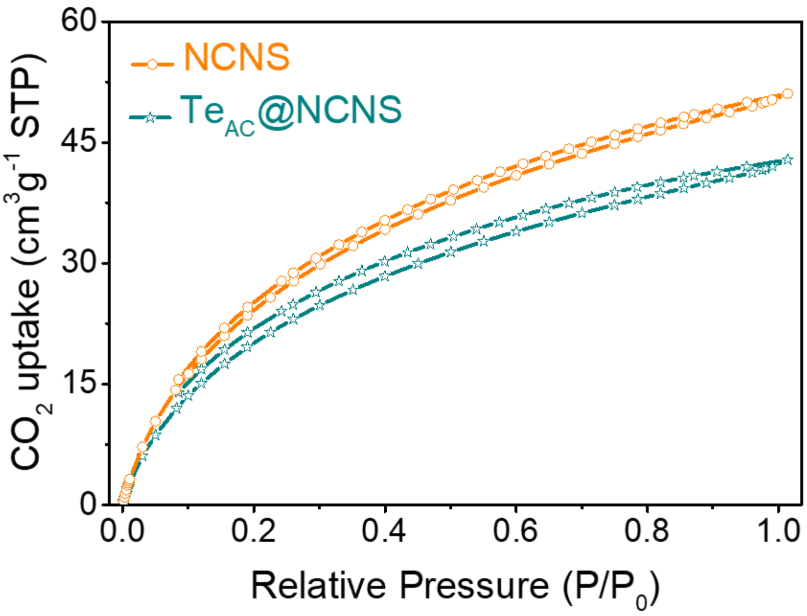

Figure S12. CO_2 adsorption isotherms of Te_{AC}@NCNS and NCNS samples at 298 K.

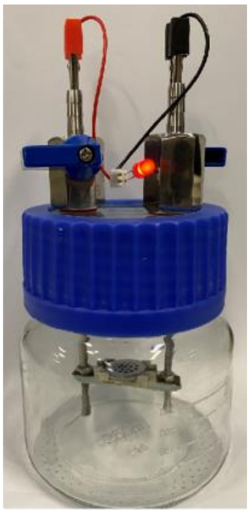

Figure S13. The home-made testing set-up for electrochemical measurements.

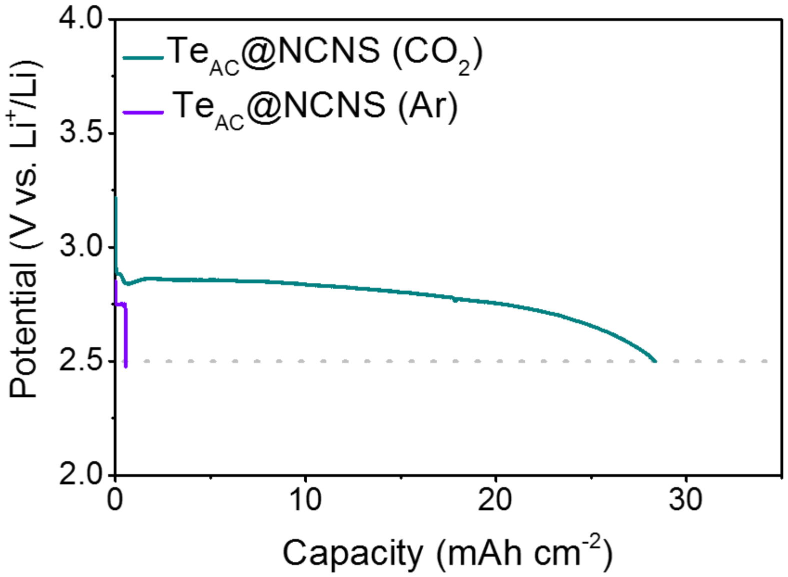

Figure S14. The first deep discharge curve of Te_{AC}@NCNS cathode was performed in Ar and CO_2 atmosphere.

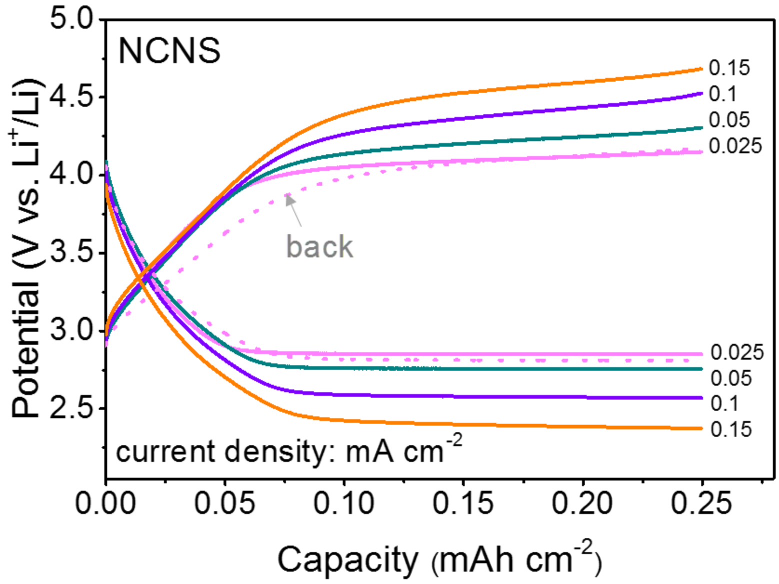

Figure S15. Discharge-charge profiles of the NCNS tested at various current densities.

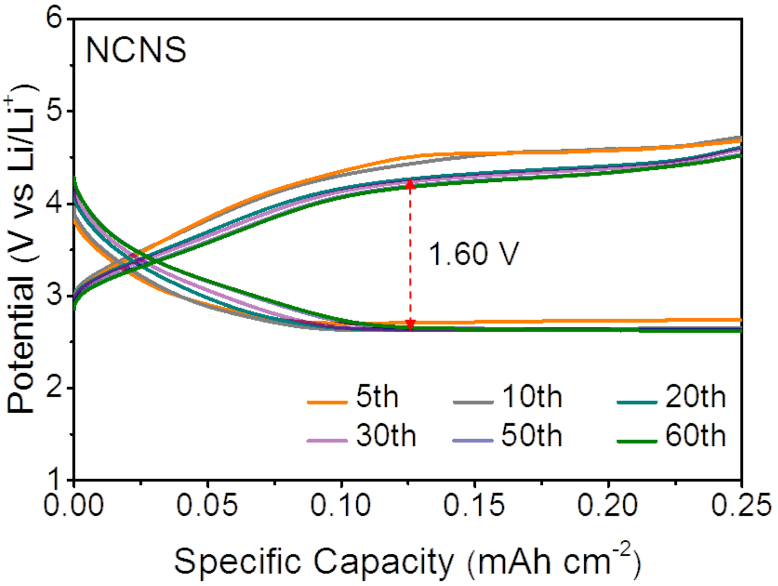

Figure S16. Discharge-charge profiles of the NCNS tested at 0.05 mA cm^{-2}.

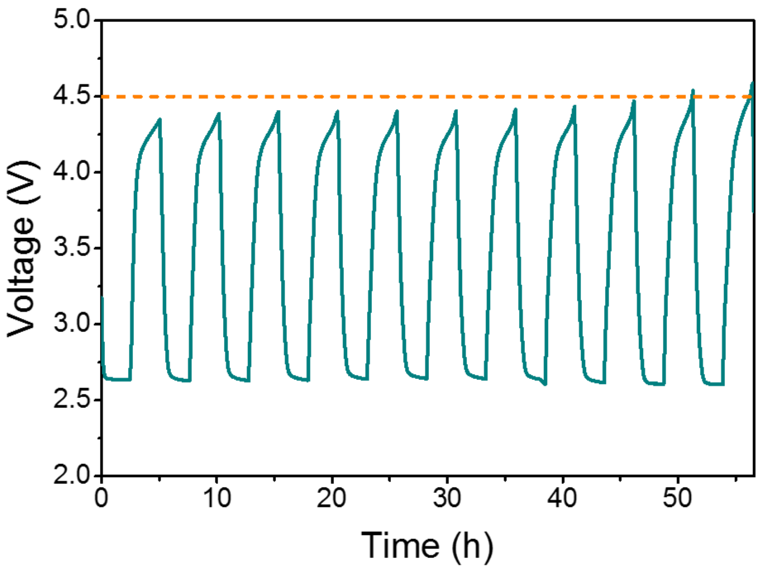

Figure S17. Cycling performance of NCNS cathode at 0.1 mA cm^{-2} and a limited capacity of 0.25 mAh cm^{-2}.

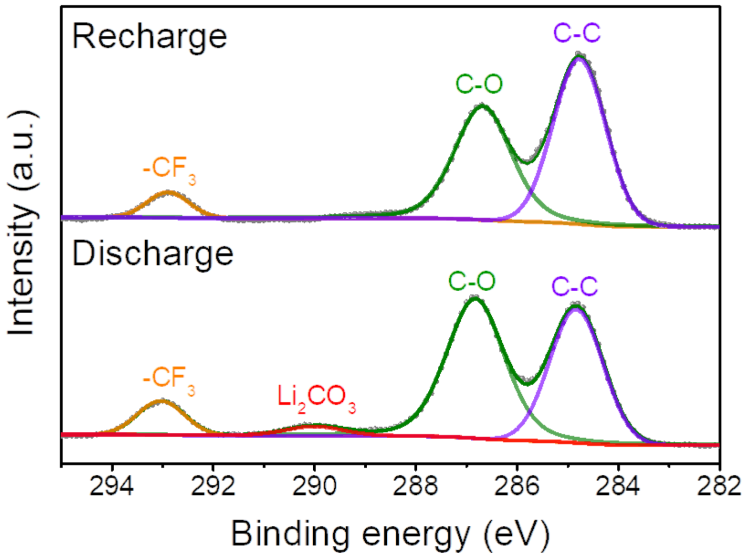

Figure S18. The high-resolution XPS spectra of C 1*s* orbits for NCNS at discharge and recharge states.

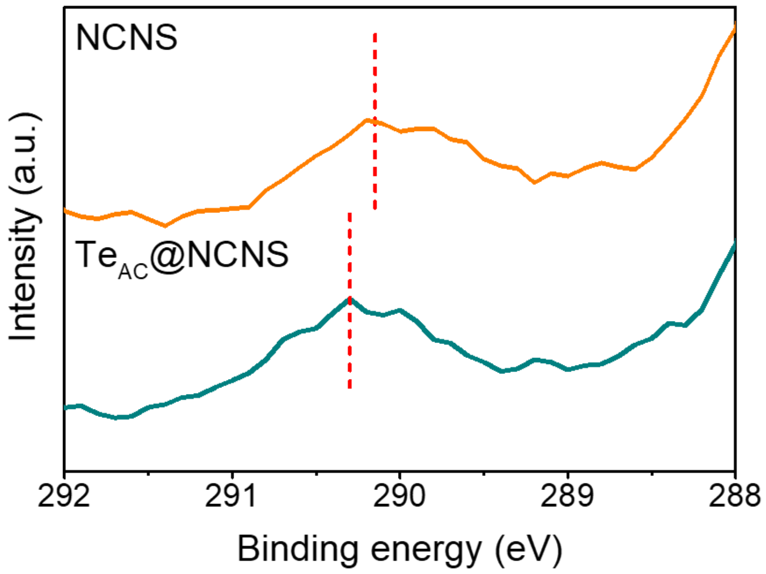

Figure S19. XPS spectra of -O-C-O- for the discharged Te_{AC}@NCNS and NCNS.

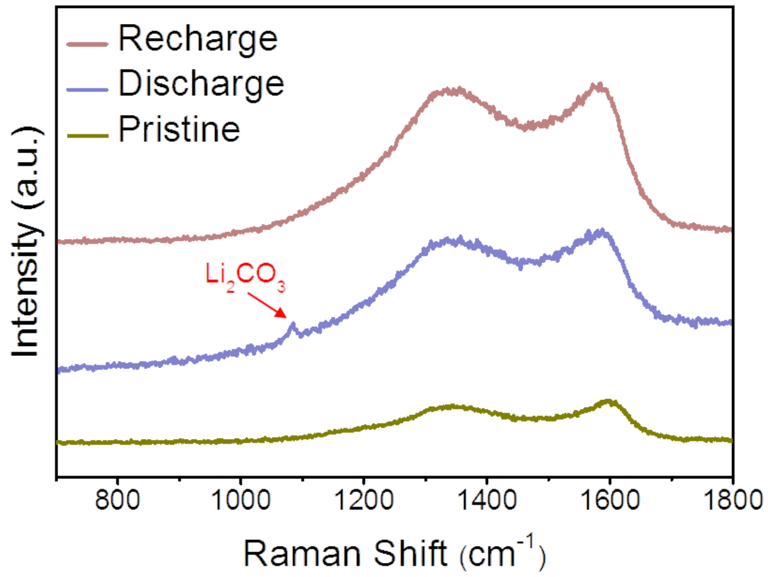

Figure S20. Raman spectra of the NCNS cathode at different stages.

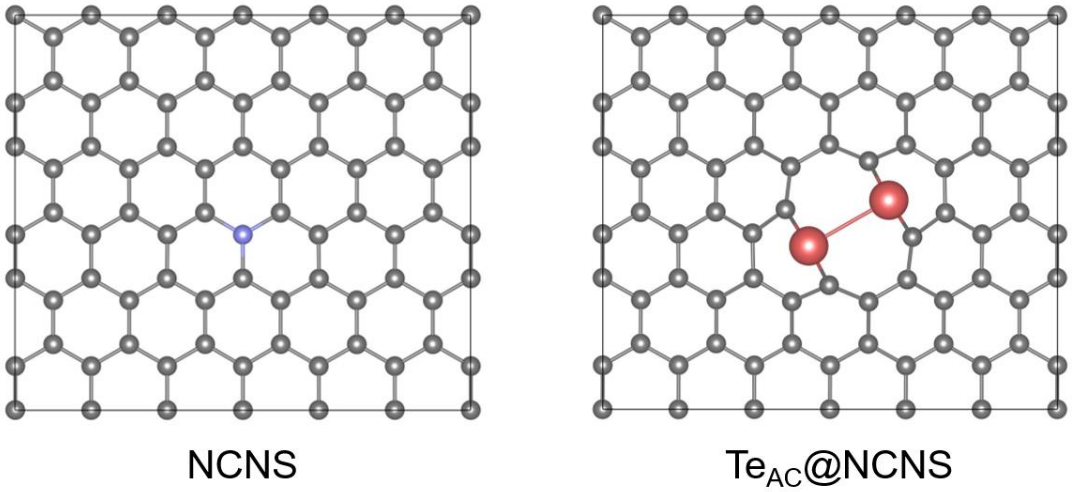

Figure S21. Top view of the NCNS and Te_{AC}@NCNS models used in DFT calculations. The N atom is not introduced with the Te cluster because the experimental results indicate Te clusters are unlikely to interplay with the N atoms. Color code: grey for C, blue for N, and red for Te.

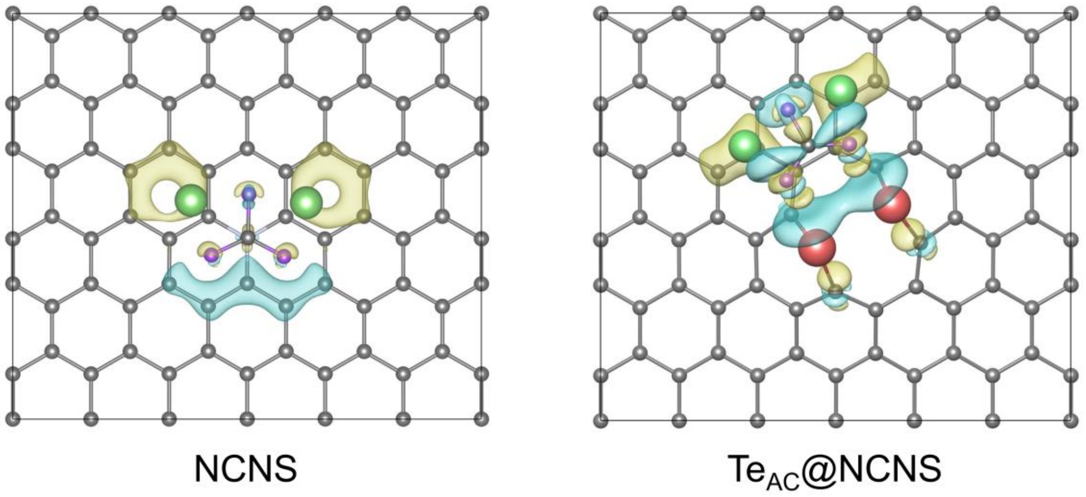

Figure S22. Top view of the charge density differences of Li_2CO_3 on NCNS (isovalue = 0.001) and Te_{AC}@NCNS (isovalue = 0.002) models. Color code: grey for C, blue for N, red for Te, purple for O, and green for Li.

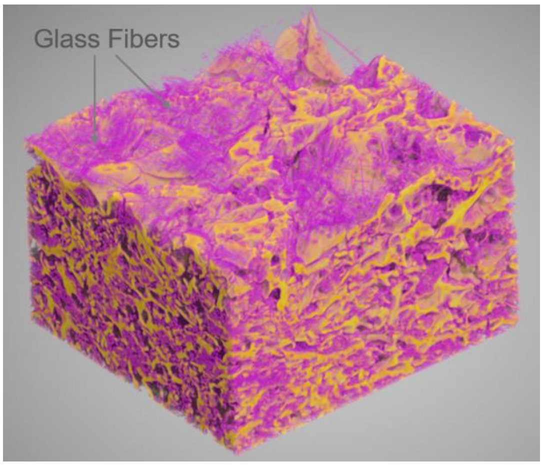

Figure S23. The three-dimensional X-ray tomography plot conducted on the Te_{AC}@NCNS cathode after discharging to 10.0 mAh cm^{-2}.

Table S1. Pore structures of NCNS and Te_{AC}@NCNS samples.

Samples	S_{BET} (m^2/g)	V_{pore} (cm^3/g)	D_{pore} (nm)
NCNS	455.35	0.164	12.219
Te_{AC}@NCNS	416.46	0.159	12.656

S_{BET} represents the BET surface area.
V_{pore} represents the volume of pores.
D_{pore} represents the average pore diameter.

As shown in Table S1, with the incorporation of Te atomic clusters, the pore volume slightly decreased, which can be attributed to the covering of the surface and some pore blocking of the support caused by Te atomic clusters. Additionally, the Te atomic clusters were anchored on the as-synthesized NCNS via chemical vapor deposition (Figure S4). During this heat treatment process, the pores of samples may collapse and merge. Generally, when the number of pores decreases and the pore

enlarges, the BET surface area decreases. As a result, after the introduction of Te atomic clusters, the BET surface area of the Te_{AC}@NCNS samples decreases slightly.

Table S2. Comparison and summary of recent cathodes on electrochemical performance for Li-CO_2 batteries.

Sample	Curtailing capacity (mAh cm^{-2})	Current density (mA cm^{-2})	Operation time /Cycle number	Overpotential (V)	Full-discharge capacity (mAh cm^{-2})
Te_{AC}@NCNS (This work)	0.25	0.05	600h/60	1.23	28.35
	0.25	0.1	600h/120	1.56	
	1	0.05	1000h/25	1.51	
s-Ir/NCNTs[2]	0.18	0.018	2400h/120	1.07	NA
RuCo NSs/CNT[3]	0.18-0.27	0.018-0.027	860h/43	0.94	1.43-2.14
CIS/CC[4]	0.1	0.02	1050h/1005	0.5-0.7	8.878
Holey CNTs[5]	0.09	0.018	1500h/150	1.18	3.15
Ru_h-NC@rGO[6]	0.2	0.2	734h/367	~2.0	7.3
0.2Cu-Co_4N@CC[7]	0.13-0.22	0.052-0.088	655h/131	1.3	4.22-7.03
Ru@CCA[8]	0.1	0.02	1720h/172	0.85	10.71
CC@MoN NFs[9]	0.1	0.01	540h/27	0.36	6.542
CCGA[10]	2.27	0.227	2000h/100	1.53	17.880
IrO_2-N/CNT[11]	0.08-0.12	0.02-0.03	2528h/316	1.34	0.93-1.39
Ru/Ni[12]	0.15	0.015	1000h/100	~1.2	1.4253
Ru_{AC+SA}@NCB[13]	0.1-0.175	0.06-0.105	200h/60	~1.3	2.13-3.73
CNT@RuO_2[14]	0.15-0.25	0.015-0.025	1100h/55	~1.36	1.16-1.93
BN-hG[15]	0.3	0.3	400h/200	1.7	4.498
CNTs[16]	0.27-0.45	0.013-0.022	600h/15	1.86	2.24-3.74
CQD/hG[17]	~0.05	0.105	240h/235	1.45	1.294
TDG[18]	~0.08	0.157	600h/600	1.6	11.018
Fe NPs@N-CNT[19]	0.210	0.035	660h/55	~1.65	1.36
N-CNTs@Ti[20]	0.45	0.112	360h/45	1.93	4.181
ZnS/N-rGO[21]	0.2-0.5	0.08-0.2	950h/190	1.52	2.06-5.16
Cu-NG[22]	0.27-0.44	0.054-0.088	500h/50	0.77	3.94-6.57
MNT[23]	0.5	0.1	1000h/100	1.38	5.729
W_2C-CNTs[24]	0.3	0.03	400h/20	~0.5	3.189
Fe-ISA/N,S-HG[25]	0.21	0.21	420h/210	1.75	4.878
CoPPc[26]	1	0.05	2000h/50	1.08	13.6

Note: All the above electrochemical data were got directly from the curves in

literatures or converted/evaluated on the basis of the given electrochemical performance curves. And “NG” represents that the data couldn’t obtain from the literatures by any aforementioned method.

References

1. Pan F., et al. Promoting electrocatalytic CO_2 reduction on nitrogen-doped carbon with sulfur addition. Appl. Catal. B 2019. **252**, 240-249.
2. Rho Y.-J., et al. Atomically miniaturized bi-phase IrOx/Ir catalysts loaded on N-doped carbon nanotubes for high-performance Li-CO_2 batteries. J Mater. Chem. A, 2022, **10**, 19710-19721.
3. Wang Y., et al. Decreasing the Overpotential of Aprotic Li-CO_2 Batteries with the In-Plane Alloy Structure in Ultrathin 2D Ru-Based Nanosheets. Adv. Funct. Mater., 2022. **32**, 2202737.
4. Chen L., et al. Copper Indium Sulfide Enables Li-CO_2 Batteries with Boosted Reaction Kinetics and Cycling Stability. Energy. Environ. Mater., 10.1002/eem2.12415.
5. Xie H., et al. Boosting Li-CO_2 battery performances by creating holey structure on CNT cathodes. Electrochimi. Acta, 2022. **417**, 140310.
6. Cheng J., et al. Homogenizing Li_2CO_3 Nucleation and Growth through High-Density Single-Atomic Ru Loading toward Reversible Li-CO_2 Reaction. ACS Appl. Mater. Inter., 2022. **14**(16), 18561-18569.
7. Ma X., et al. In-situ construction of Cu-Co_4N@CC hierarchical binder-free cathode for advanced and flexible Li-CO_2 batteries: Electron structure and mass transfer modulation. J. Power Sources, 2022. **535**, 231446.
8. Liu L., et al. Rational Design of Nanostructured Metal/C Interface in 3D Self-Supporting Cellulose Carbon Aerogel Facilitating High-Performance Li-CO_2 Batteries. Adv. Energy Mater., 2022. **12**(20), 2103681.
9. Qi G., et al. Binder-Free MoN Nanofibers Catalysts for Flexible 2-Electron Oxalate-Based Li-CO_2 Batteries with High Energy Efficiency. Adv. Funct. Mater., 2022. **32**(22), 2112501.
10. Deng Q., et al. Electron structure and reaction pathway regulation on porous cobalt-doped CeO_2/graphene aerogel: A free-standing cathode for flexible and advanced Li-CO_2 batteries. Energy Storage Mater., 2021. **42**, 484-492.
11. Wu G., et al. Design of ultralong-life Li-CO_2 batteries with IrO_2 nanoparticles highly dispersed on nitrogen-doped carbon nanotubes. J. Mater. Chem. A, 2020. **8**(7), 3763-3770.
12. Zhao H., et al. Ru nanosheet catalyst supported by three-dimensional nickel foam as a binder-free cathode for Li-CO_2 batteries. Electrochimica Acta, 2019. **299**, 592-599.
13. Lin J., et al. Boosting Energy Efficiency and Stability of Li-CO_2 Batteries via Synergy between Ru Atom Clusters and Single-Atom Ru-N4 sites in the Electrocatalyst Cathode. Adv. Mater. 2022. **34**(17), 2200559.
14. Bie S., et al. Carbon Nanotube@RuO_2 as a High Performance Catalyst for Li-CO_2 Batteries. ACS Appl. Mater. Inter., 2019. **11**(5), 5146-5151.
15. Qie L., et al. Highly Rechargeable Lithium-CO_2 Batteries with a Boron- and Nitrogen-Codoped Holey-Graphene Cathode. Angew. Chem. Int. Ed. 2017. **56**(24), 6970-6974.
16. Li S., et al. Carbon dioxide in the cage: manganese metal-organic frameworks for high performance CO2 electrodes in Li-CO_2 batteries. Energy Environ. Sci., 2018. **11**(5),

1318-1325.
17. Jin Y., et al. High-Performance Li-CO_2 Batteries Based on Metal-Free Carbon Quantum Dot/Holey Graphene Composite Catalysts. Adv. Funct. Mater., 2018. **28**(47), 1804630.
18. Ye F., et al. Topological Defect-Rich Carbon as a Metal-Free Cathode Catalyst for High-Performance Li-CO_2 Batteries. Adv. Energy Mater., 2021. **11**, 2101390.
19. Kim H.-S., et al. Capillary-Driven Formation of Iron Nanoparticles Embedded in Nanotubes for Catalyzed Lithium-Carbon Dioxide Reaction. ACS Materials Letters, 2021. **3**(6), 815-825.
20. Li Y., et al. Highly Surface-Wrinkled and N-Doped CNTs Anchored on Metal Wire: A Novel Fiber-Shaped Cathode toward High-Performance Flexible Li-CO_2 Batteries. Adv. Funct. Mater., 2019. **29**(12), 1808117.
21. Wang H., et al. Realizing Interfacial Electronic Interaction within ZnS Quantum Dots/N-rGO Heterostructures for Efficient Li-CO_2 Batteries. Adv. Energy Mater., 2019. **9**(34), 1901806.
22. Zhang Z., et al. Identification of cathode stability in Li-CO_2 batteries with Cu nanoparticles highly dispersed on N-doped graphene. Journal of Materials Chemistry A, 2018. **6**(7), 3218-3223.
23. Hu Z., et al. Hierarchical $Ti_3C_2T_x$ MXene/Carbon Nanotubes for Low Overpotential and Long-Life Li-CO_2 Batteries. ACS Nano, 2021. **15**(5), 8407-8417.
24. Zhang X., et al. Breaking the Stable Triangle of Carbonate via W-O Bonds for Li-CO_2 Batteries with Low Polarization. ACS Energy Letters, 2021. **6**(10), 3503-3510.
25. Hu C., et al. High-Performance, Long-Life, Rechargeable Li-CO_2 Batteries based on a 3D Holey Graphene Cathode Implanted with Single Iron Atoms. Adv. Mater., 2020. **32**(16), 1907436.
26. Chen J., et al. Conjugated Cobalt Polyphthalocyanine as the Elastic and Reprocessable Catalyst for Flexible Li-CO_2 Batteries. Adv. Mater., 2019. **31**(2), 1805484.
